# Pediatric COVID-19 Hospitalization Trends by Race and Ethnicity, 2020–2023

**DOI:** 10.1001/jamanetworkopen.2025.21009

**Published:** 2025-07-01

**Authors:** Onika Anglin, Kadam Patel, Pam Daily Kirley, Darpun Sachdev, Nisha B. Alden, Isaac Armistead, Kimberly Yousey-Hindes, James Meek, Lucy S. Witt, Kyle P. Openo, Maya L. Monroe, Sue Kim, Elizabeth Urlaub, Kathryn Como-Sabetti, Paige D’Heilly, Susan L. Ropp, Nancy Eisenberg, Jemma V. Rowlands, Grant Barney, Sophrena Bushey, Katherine St. George, Melissa Sutton, Nasreen Abdullah, William Schaffner, H. Keipp Talbot, Ryan Chatelain, Andrea Price, Hope King, Christopher A. Taylor, Monica E. Patton, Fiona P. Havers

**Affiliations:** National Center for Immunization and Respiratory Diseases, Centers for Disease Control and Prevention, Atlanta, Georgia (Anglin, Patel, King, Taylor, Patton, Havers); California Emerging Infections Program, Oakland (Daily Kirley); California Department of Public Health, Richmond (Sachdev); Colorado Department of Public Health & Environment, Denver (Alden, Armistead); Connecticut Emerging Infections Program, Yale School of Public Health, New Haven (Yousey-Hindes, Meek); Division of Infectious Diseases, Emory University School of Medicine, Atlanta, Georgia (Witt, Openo); Georgia Emerging Infections Program, Georgia Department of Public Health, Atlanta (Witt, Openo); Research, Atlanta Veterans Affairs Medical Center, Decatur, Georgia (Openo); Maryland Department of Health, Baltimore (Monroe); Michigan Department of Health and Human Services, Lansing (Kim, Urlaub); Minnesota Department of Health, St Paul (Como-Sabetti, D’Heilly); New Mexico Department of Health, Santa Fe (Ropp); University of New Mexico Emerging Infections Program, Albuquerque (Eisenberg); New York State Department of Health, Albany (Rowlands, Barney); University of Rochester School of Medicine and Dentistry, Rochester, New York (Bushey, St. George); Public Health Division, Oregon Health Authority, Portland (Sutton, Abdullah); Vanderbilt University Medical Center, Nashville, Tennessee (Schaffner, Talbot); Salt Lake County Health Department, Salt Lake City, Utah (Chatelain, Price); United States Public Health Service, Rockville, Maryland (Patton, Havers).

## Abstract

**IMPORTANCE:**

Examining racial and ethnic disparities in pediatric COVID-19 hospitalizations is critical to inform public health efforts to reduce those disparities.

**OBJECTIVE:**

To characterize trends in pediatric COVID-19 hospitalizations by race and ethnicity from March 2020 to September 2023, focusing on recent epidemiologic findings (October 2022 to September 2023).

**DESIGN, SETTING, AND PARTICIPANTS:**

This cross-sectional study used data from the COVID-19 Hospitalization Surveillance Network (COVID-NET) including 13 555 hospitalizations among patients aged 17 years or younger with a laboratory-confirmed SARS-CoV-2 infection who are residents of the COVID-NET catchment area in 12 states, covering approximately 10% of the US population.

**EXPOSURE:**

Laboratory-confirmed SARS-CoV-2 infection within 14 days prior to or during hospitalization.

**MAIN OUTCOMES AND MEASURES:**

Pediatric COVID-19–associated hospitalization rates by race and ethnicity and characteristics associated with COVID-19–associated hospitalizations.

**RESULTS:**

Between March 2020 and September 2023, COVID-NET identified 13 555 pediatric hospitalizations (median patient age, 3.3 years [IQR, 0.6–12.5 years]; 7110 boys [52.5%]; 780 non-Hispanic Asian or Pacific Islander children [5.8%], 3837 non-Hispanic Black children [28.3%], 4131 Hispanic children [30.5%], and 4807 non-Hispanic White children [35.5%]). Hospitalization rates were 2.15 (95% CI, 2.01–2.34) times higher for Black children and 2.06 (95% CI, 1.91–2.23) times higher for Hispanic children compared with Asian or Pacific Islander children, who had the lowest rates. Despite overall decreased pediatric hospitalization rates between October 2022 and September 2023, higher rates of intensive care unit admissions among Black and Hispanic children persisted, at 1.88 (95% CI, 1.28–2.74) times higher for Black children and 2.13 (95% CI, 1.47–3.10) times higher for Hispanic children compared with Asian or Pacific Islander children. Among hospitalized children, 61.4% (95% CI, 57.0%−65.8%) of Black patients and 45.5% (95% CI, 41.9%−49.3%) of Hispanic patients had 1 or more underlying medical condition compared with 45.6% (95% CI, 42.1%−49.1%) of White children and 45.0% (95% CI, 41.9%−49.3%) of Asian or Pacific Islander children. Obesity (17.8%; 95% CI, 15.3%−20.5%) and neurologic disorders (15.2%; 95% CI, 13.7%−16.8%) were the most common conditions overall; 11.9% (95% CI, 9.1%−15.1%) of Black children had sickle cell disease, the fourth most common condition in this group.

**CONCLUSIONS AND RELEVANCE:**

This study found that among pediatric patients hospitalized with COVID-19, Black and Hispanic children were disproportionately more likely to be hospitalized for COVID-19 and experience severe disease compared with White and Asian or Pacific Islander children. A higher proportion of hospitalized Black children had underlying medical conditions. This study underlines the need for targeted interventions, particularly for children with underlying medical conditions, and the need for equitable access and use of vaccines and therapeutics for disproportionately affected populations.

## Introduction

Since the start of the COVID-19 pandemic in 2020, most hospitalizations for COVID-19 have occurred among adults,^1^ and multiple studies demonstrated that COVID-19 hospitalizations disproportionately affected and exacerbated existing health disparities among adults of racial and ethnic minority populations, with hospitalization rates more than 5-fold higher^2^ among Black and Hispanic populations compared with White adult populations in 2020. However, COVID-19 also causes severe illness in infants and children,^3,4^ but information is lacking on racial and ethnic disparities in COVID-19 among pediatric patients.^5–8^ Although a 2022 report demonstrated that disparities in hospitalizations by race and ethnicity also occurred among pediatric populations,^9^ few studies have examined these disparities over the course of the pandemic. This analysis examines observed racial and ethnic disparities and outcomes among children hospitalized with COVID-19 from March 2020 to September 2023.

## Methods

The COVID-19 Hospitalization Surveillance Network (COVID-NET) is a population-based active surveillance system that captures laboratory-confirmed COVID-19–associated hospitalizations from 90 counties in 12 states (California, Colorado, Connecticut, Georgia, Maryland, Michigan, Minnesota, New Mexico, New York, Oregon, Tennessee, and Utah), covering approximately 10% of the US population. COVID-NET cases have a clinician-ordered positive SARS-CoV-2 test result within 14 days prior to or during hospitalization and are residents of the defined catchment area. Patient consent was not required as COVID-NET data used in this analysis were collected as part of activities reviewed by the Centers for Disease Control and Prevention (CDC) and determined to be collected as part of public health surveillance. Activities were conducted consistent with applicable federal law and CDC policy (45 CFR part 46.102[l][2], 21 CFR part 56; 42 USC §241[d]; 5 USC §552a; 44 USC §3501 et seq). Sites participating in COVID-NET obtained approval from their respective state and local institutional review boards, as applicable. This study adhered to the Strengthening the Reporting of Observation Studies in Epidemiology (STROBE) reporting guidelines.

This analysis includes COVID-NET data from children aged 17 years or younger residing in the catchment area who were hospitalized from March 2020 through September 2023. This period was divided into 4 surveillance periods spanning October to September except for the first year: March to September 2020, October 2020 to September 2021, October 2021 to September 2022, and October 2022 to September 2023. Demographic data were obtained on all COVID-NET patients and were used to calculate hospitalization rates by race and ethnicity in the following categories: non-Hispanic Asian or Pacific Islander (hereafter, *Asian or Pacific Islander*), non-Hispanic Black (hereafter, *Black*), Hispanic or Latino (hereafter, *Hispanic*), and non-Hispanic White (hereafter, *White*), where Hispanic persons could be of any race. If race was known but ethnicity was unknown, cases were classified as non-Hispanic. Race and ethnicity were obtained from the medical records, notifiable disease databases, laboratory data, and hospital databases as reported by the patient, parent, or guardian; patients with missing or unknown race were excluded from this analysis. American Indian or Alaska Native children and children of multiple races accounted for a disproportionate burden of hospitalizations in comparison with population size. However, these groups were excluded because case counts were too low to produce stable population-based rates.

To assess COVID-19–associated hospitalizations among specific pediatric racial and ethnic groups, cumulative hospitalization rates were calculated overall (aged ≤17 years) and by pediatric age group (≤4, 5–11, and 12–17 years) for the entire period (March 2020 to September 2023). To examine trends across multiple periods, hospitalization and intensive care unit (ICU) admission rates were calculated by period and race and ethnicity. National Center for Health Statistics vintage 2020 bridged-race postcensal population estimates (March-September 2020) or US Census vintage unbridged-race postcensal population estimates^10^ (October 2020 to September 2023) for catchment counties were used as denominators to calculate hospitalization and ICU admission rates per 100 000 population with 95% CIs. The 95% CIs were calculated by Taylor series linearization. The group with the lowest hospitalization rates, Asian or Pacific Islander, was used as the reference group for rate ratios.

To better characterize disparities in severe COVID-19 illness among specific racial and ethnic groups that reflect recent COVID-19 epidemiologic findings, hospitalization and ICU rates by age and racial and ethnic group were examined for October 2022 to September 2023. In addition to demographic data on all patients, patient outcomes, ICU admissions, in-hospital deaths, and detailed clinical data, including underlying medical conditions, clinical outcomes, and vaccination data, were captured through medical record review of an age- and site-stratified random sample of all hospitalized patients performed by trained surveillance officers using previously established methods.^11^ Sampling weights were applied monthly to account for selection probability and varied by month, site, and pediatric age group.^11^ Percentages shown in these data are weighted to account for probability of selection to obtain a representative sample and were further adjusted to account for nonresponse (eg, an incomplete medical record review). The COVID-19 vaccination status of hospitalized children from October 2022 to September 2023 was ascertained through state immunization information systems or patient medical records. A patient was assigned an up-to-date vaccination status if they received the most recent age-specific recommended COVID-19 vaccine at least 14 days prior to a positive SARS-CoV-2 test result.^12^ Children aged younger than 6 months were not eligible to receive the COVID-19 vaccine and were excluded from these calculations. COVID-19 vaccination eligibility changed by age group over time during the study period. Vaccine-eligible children and adolescents aged 6 months or older without any evidence of ever having received a COVID-19 vaccination were categorized as having no record of COVID-19 vaccination. Before the 2022–2023 (bivalent) formula doses were recommended, children and adolescents who completed a COVID-19 primary vaccination series 14 days or more before the positive SARS-CoV-2 test result associated with their hospitalization were categorized as up to date. After the 2022–2023 booster doses were recommended, only those who completed the COVID-19 primary series and subsequently received a recommended booster dose 14 days or more before testing positive for SARS-CoV-2 were categorized as up to date. Children and adolescents who began, but did not complete, a COVID-19 primary vaccination series, or who completed a primary series but did not receive a recommended booster dose 14 days or more before testing positive for SARS-CoV-2 were categorized as having received 1 dose or more of COVID-19 vaccine but were not considered up to date. Analysis of clinical data was limited to hospitalizations where the presenting complaint was likely COVID-19–related illness and excluded those hospitalized primarily for inpatient surgery or procedures, psychiatric admissions, obstetrics, labor and delivery, or trauma, as determined by a review of the chief complaint, presenting symptoms, and admission history.^13^

### Statistical Analysis

Rates were presented per 100 000 children. Categorical differences between groups were compared using χ^2^ tests, with *P* < .05 considered statistically significant. Differences in group-specific characteristics, including age distribution, ICU status, insurance status, underlying medical conditions, and vaccination status, were compared using univariate linear regression. A 2-tailed *P* < .05 was considered statistically significant. Rate ratios with a 95% CI that excluded 1 were considered statistically significant. In addition, 95% CIs were provided for weighted percentages. Data analyses were conducted using SAS, version 9.4 (SAS Institute Inc).

## Results

Of 14 661 pediatric COVID-19–associated hospitalizations reported from March 2020 to September 2023, 1106 (7.5%) were excluded from rate calculations because race or ethnicity was unknown (727 of 1106 [4.9%]) or the patient was identified as multiracial or American Indian or Alaska Native (379 of 1106 [2.6%]) (eFigure 1 in Supplement 1; characteristics of excluded children shown in eTable in Supplement 1). A total of 13 555 pediatric hospitalizations (median patient age, 3.3 years [IQR, 0.6–12.5 years]; 7110 boys [52.5%] and 6445 girls [47.5%]) were included for rate calculations and comprised 780 Asian or Pacific Islander children (5.8%), 3837 Black children (28.3%), 4131 Hispanic children (30.5%), and 4807 White children (35.5%) (Table 1). Black and Hispanic children comprised 58.8% (n = 7968) of cases, while representing 40.3% of the pediatric catchment population.

**Figure 1.**
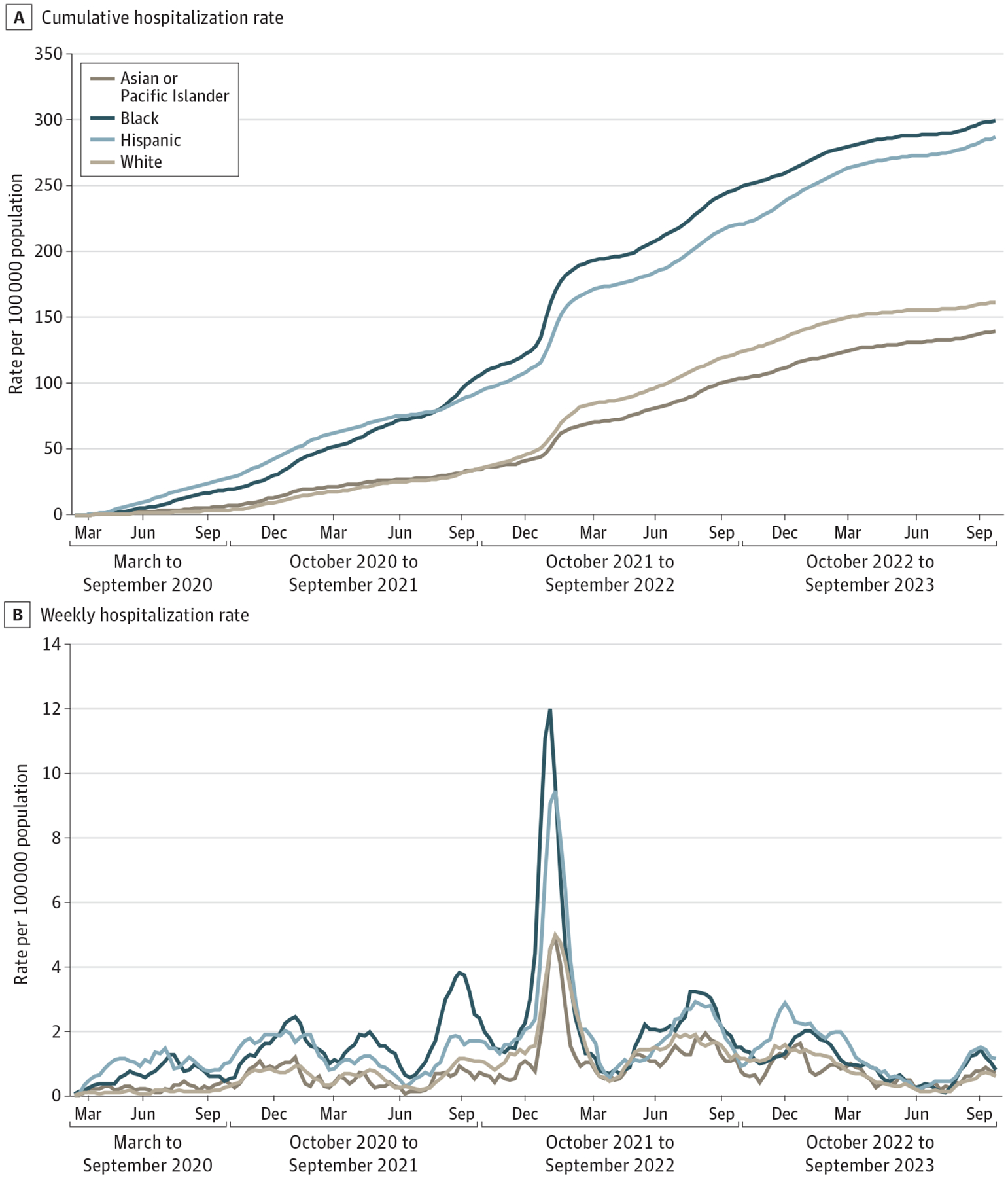
Pediatric COVID-19 Hospitalization Rates by Race and Ethnicity and Respiratory Virus Surveillance Period, March 2020 to September 2023 A, Cumulative pediatric COVID-19 hospitalization rates by race and ethnicity among patients aged 17 years or younger. B, Weekly pediatric COVID-19 hospitalization rates (3-week moving mean) by race and ethnicity among patients aged 17 years or younger.

Cumulative and weekly hospitalization rates from March 2020 to September 2023 overall indicated that Hispanic and Black children were disproportionately hospitalized compared with their Asian or Pacific Islander and White peers (Figure 1A and 1B). COVID-19–associated hospitalization rates were consistently highest among Black and Hispanic children aged 17 years or younger (Figure 1A and 1B) and among all age groups (eFigure 2A–C in Supplement 1). Overall hospitalization rates were 2.15 (95% CI, 2.01–2.34) times greater among Black children and 2.06 (95% CI, 1.91–2.23) times greater among Hispanic children compared with Asian or Pacific Islander children. Although hospitalization rates were lower among children aged 5 to 11 years and 12 to 17 years compared with children aged 4 years or younger, there were greater disparities by race and ethnicity among older children compared with those aged 4 years or younger (eFigure 2A–C in Supplement 1). Among children aged 4 years or younger, cumulative hospitalization rates were 1.64 (95% CI, 1.50–1.83) times greater for Black children and 1.74 (95% CI, 1.60–1.95) times greater for Hispanic children compared with Asian or Pacific Islander children; these rate ratios increased to 2.82 (95% CI, 2.36–3.47) times greater for Black children and 2.37 (95% CI, 1.992.93) times greater for Hispanic children among those aged 5 to 11 years and increased to 3.20 (95% CI, 2.71–3.74) times greater for Black children and 2.66 (95% CI, 2.23–3.07) times greater for Hispanic children among those aged 12 to 17 years. Rate ratios for White children of all ages compared with Asian or Pacific Islander children were not as disparate: 1.06 (95% CI, 1.02–1.24) for children aged 4 years or younger, 1.35 (95% CI, 1.15–1.69) for those aged 5 to 11 years, and 1.41 (95% CI, 1.23–1.70) for those aged 12 to 17 years.

**Figure 2.**
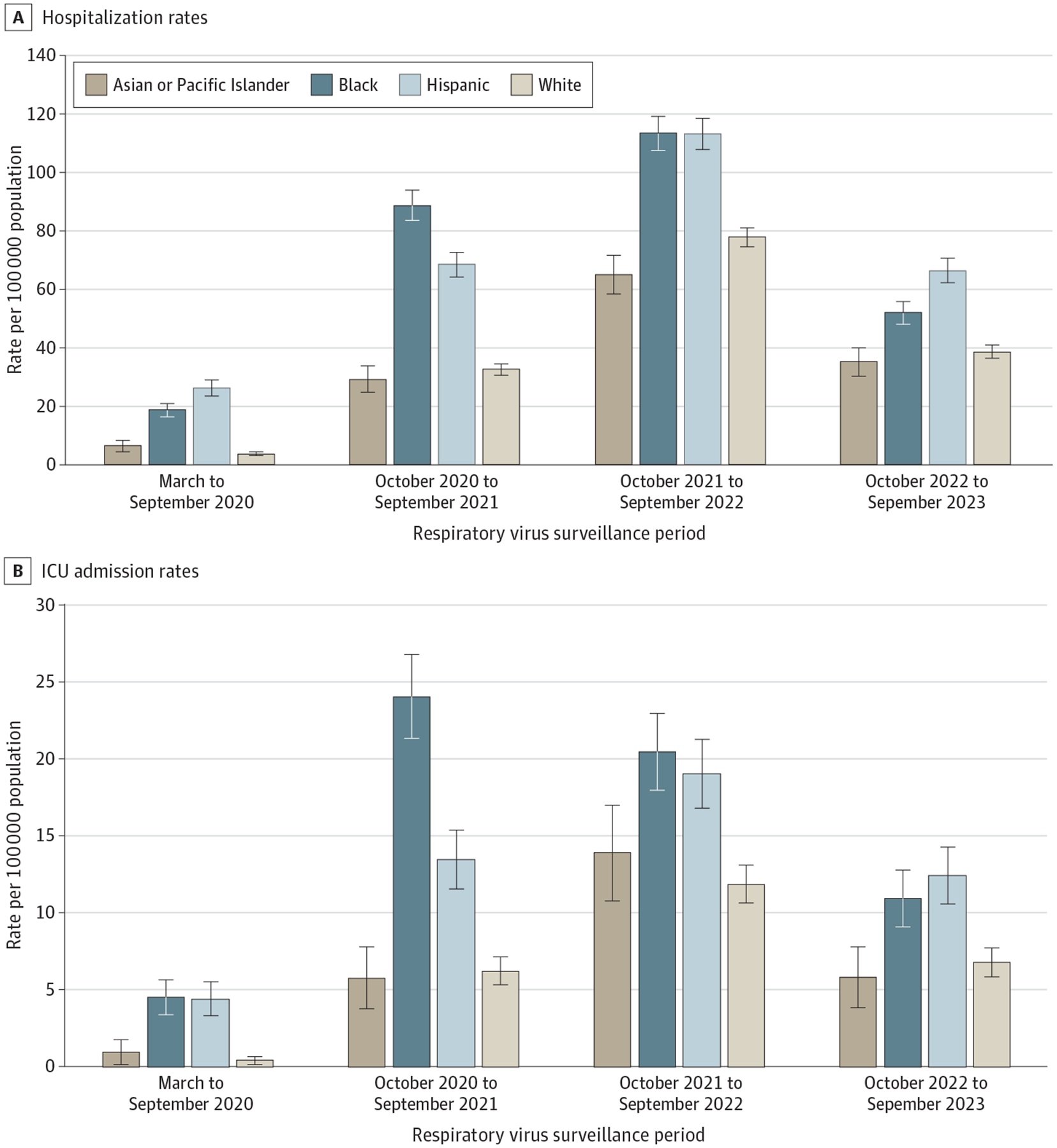
Pediatric COVID-19 Hospitalization and Intensive Care Unit (ICU) Admission Rates by Race, Ethnicity, and Respiratory Virus Surveillance Period, March 2020 to September 2023 A, Pediatric hospitalization rates by race, ethnicity, and respiratory virus surveillance period. B, Pediatric ICU admission rates by race, ethnicity, and respiratory virus surveillance period. Error bars indicate 95% CIs.

The October 2021 to September 2022 period had the highest cumulative hospitalization rates per 100 000 population among all racial and ethnic groups, although rates were significantly higher among Black (113.2; 95% CI, 107.33–118.99) and Hispanic (113.0; 95% CI, 107.48–118.43) children compared with Asian or Pacific Islander (64.8; 95% CI, 58.10–71.49) and White (77.6; 95% CI, 74.48–80.79) children (Figure 2A). In 2022 and 2023, hospitalization rates decreased among all racial and ethnic groups, and disparities narrowed. However, hospitalization rates among Black and Hispanic children remained consistently higher compared with Asian or Pacific Islander children for each period (Figure 2A).

Similarly, disparities in ICU admission rates were also observed initially and persisted throughout each period, even while ICU admission rates decreased over time (Table 2; Figure 2B). Intensive care unit admission rates for Black children were 4.14 (95% CI, 2.88–5.96) times greater from October 2020 to September 2021, 1.47 (95% CI, 1.14–1.90) times greater from October 2021 to September 2022, and 1.88 (95% CI, 1.28–2.75) times greater from October 2022 to September2023 than rates for Asian or Pacific Islander children. Intensive care unit admission rates among Hispanic children were 2.33 (95% CI, 1.60–3.38) times greater from October 2020 to September 2021, 1.37 (95% CI, 1.06–1.76) times greater from October 2021 to September 2022, and 2.13 (95% CI, 1.47–3.10) times greater from October 2022 to September 2023 than those of Asian or Pacific Islander (Figure 2B).

A total of 2911 hospitalizations were identified during the October 2022 to September 2023 period, with 742 cases excluded from the final analytic sample because no additional clinical data were captured (n = 29) or the case was admitted for a reason that was likely not COVID-19 related (n = 713; eFigure 1 in Supplement 1). Among the 2169 included in the final analytic sample of children hospitalized from October 2022 to September 2023, 152 (7.0%) were Asian or Pacific Islander, 490 (22.6%) were Black, 726 (33.5%) were Hispanic, and 801 (36.9%) were White (Table 3). Children aged 4 years or younger accounted for 74.3% (95% CI, 72.6%−76.3%) of hospitalizations from October 2022 to September 2023, with a median age of 0.9 years (IQR, 0.3–4.2 years). The age distribution differed significantly between racial and ethnic groups, with a higher proportion of Black children aged 5 to 11 years (16.6%; 95% CI, 13.5%−20.2%) compared with White children (13.1%; 95% CI, 10.9%−15.7% [*P* = .01]) and Hispanic children (13.2%; 95% CI, 10.9%−15.9% [*P* < .001]) and in the group aged 12 to 17 years (13.9%; 95% CI, 11.0%−17.3%) compared with Hispanic (10.1%; 95% CI, 8.0%−12.6% [*P* = .008]) and Asian or Pacific Islander children (9.0%; 95% CI, 5.0%−14.7% [*P* = .003]).

Among all children hospitalized for COVID-19 from October 2022 to September 2023, 61.4% (95% CI, 57.0%−65.8%) of Black patients had 1 or more underlying medical condition compared with 45.6% (95% CI, 42.1%−49.1%) of White, 45.5% (95% CI, 41.9%−49.3%) of Hispanic, and 45.0% (41.9%−49.3%) of Asian or Pacific Islander children. Among all children aged 17 years or younger hospitalized for COVID-19 from October 2022 to September 2023, the proportion of children with 2 or more underlying conditions was highest among Black children (30.7%; 95% CI, 26.6%−35.0%) compared with Hispanic (22.2%; 95% CI, 19.3%−25.4% [*P* < .001]), White (21.1%; 95% CI, 18.3%−24.1% [*P* < .001]), and Asian or Pacific Islander (22.1%; 95% CI, 15.8%−29.6% [*P* = .004]) children. Overall, obesity (17.8%; 95% CI, 15.3%−20.5%), neurologic disorders (15.2%; 95% CI,13.7%−16.8%), and asthma (13.5%; 95% CI, 12.1%−15.0%) were the most common conditions among children from all race and ethnicity groups. Although no statistically significant difference in obesity and neurologic disorders was observed across all race and ethnicity groups, the prevalence of asthma among hospitalized patients was higher among Black children (19.5%; 95% CI, 16.1%−23.3%) compared with Hispanic (12.2%; 95% CI, 9.9%−14.8% [*P* = .01]) and White (11.5%; 95% CI, 9.4%−13.9% [*P* = .002]) children. Sickle cell disease was the fourth most frequently identified underlying condition among Black children, reported in 11.9% (95% CI, 9.1%−15.1%) of hospitalized Black children with COVID-19.

From October 2022 to September 2023, 24.9% (95% CI, 23.1%−26.8%) of children were admitted to the ICU, among whom 35.7% (95% CI, 31.7%−39.9%) were White, 32.8% (95% CI, 28.9%−36.9%) were Hispanic, 25.5% (95% CI, 21.9%−29.4%) were Black, and 6.0% (95% CI, 4.2%−8.3%) were Asian or Pacific Islander. The proportion of hospitalized children admitted to the ICU was similar among Black, (28.3%; 95% CI, 24.4%−32.5%), Hispanic (24.2%; 95% CI, 21.1%−27.5%), White (24.1%; 95% CI, 21.2%−27.4%), and Asian or Pacific Islander children (21.5%; 95% CI, 15.2%−28.8) (*P* = .22) (Table 2), suggesting pediatric patients of all races and ethnicities have similar risk of ICU admission once hospitalized, although rates of initial COVID-19–associated hospitalization overall were disproportionately higher among Black and Hispanic children. During this period, in-hospital deaths were rare and occurred in less than 1% of pediatric hospitalizations.

Among sampled cases, nearly half (49.7%; 95% CI, 47.6%−51.9%) had private insurance, 47.3% (95% CI, 45.2%−49.5%) had Medicaid, and 2.9% (95% CI, 2.3%−3.8%) were uninsured (Table 3). A higher proportion of Black (58.0%; 95% CI, 53.5%−62.5%) and Hispanic (68.5%; 95% CI, 64.9%−71.9%) children had Medicaid coverage compared with White (22.5%; 95% CI, 19.6%−25.6%) and Asian or Pacific Islander (41.1%; 95% CI, 33.%3–49.5%) children (*P* < .001). Hispanic children had the largest proportion of uninsured children (5.1%; 95% CI, 3.6%−7.0% [*P* < .001]). Overall, the proportion of children hospitalized for COVID-19 from October 2022 to September 2023 who were vaccinated was low for all racial and ethnic groups; 4.8% (95% CI, 3.8%−6.0%) of all children aged 6 months or older had received the most recently recommended vaccine for this period respective to their age group and were considered up to date, with no statistically significant differences between racial and ethnic groups.

## Discussion

In this cross-sectional study of a large, geographically diverse, population-based surveillance system using data from more than 13 000 hospitalizations among children with laboratory-confirmed SARS-CoV-2 from the start of the COVID-19 pandemic through September 2023, Black and Hispanic children were disproportionately affected by COVID-19 hospitalizations compared with White and Asian or Pacific Islander children. These disparities were evident early in the pandemic and persisted over multiple years. Although hospitalization and ICU admission rates decreased during the period from October 2022 to September 2023 compared with earlier surveillance periods, both were highest among Black and Hispanic children, with rates nearly twice those among Asian or Pacific Islander children. Higher proportions of hospitalized Black children had underlying medical conditions compared with Asian or Pacific Islander children, with a particularly high prevalence of asthma and sickle cell disease identified among hospitalized Black children. Despite the widespread availability of COVID-19 vaccines during this period, nearly all vaccine-eligible children (95.2%) of all races and ethnicities hospitalized for COVID-19 had not received the recommended vaccine, indicating the need for increased prevention efforts, including in communities that continue to be most affected by severe COVID-19 disease.

Most literature focused on COVID-19 racial and ethnic disparities focuses on adults,^5,6,14^ with sparse literature available on pediatric hospitalizations.^3,15^ Our study confirms that Black and Hispanic children were also disproportionately affected during the first years of COVID-19 circulation (March 2020 to September 2023). Overall hospitalizations and ICU admission rates decreased between October 2021 to September 2022 and October 2022 to September 2023 among all children, as did rate ratios that compared hospitalization rates among Black and Hispanic children with Asian or Pacific Islander children. Nevertheless, disparities, while smaller, persisted, and when looking specifically at rates of severe illness, disparities for Black and Hispanic children increased in comparison with Asian or Pacific Islander children between October 2021 to September 2022 and October 2022 to September 2023. Intensive care unit admission rate ratios increased from 1.47 to 1.88 for Black children and from 1.37 to 2.13 for Hispanic children compared with their Asian or Pacific Islander peers between those 2 periods, adding to existing literature demonstrating that Black and Hispanic persons continued to be disproportionately affected by severe health outcomes.^16–19^ With increased population-level immunity as more people were vaccinated or infected with COVID-19,^20^ underlying medical conditions have played an increasingly important role as risk factors for severe pediatric COVID-19 illness,^21^ particularly among older children.^22^ This finding, coupled with a higher prevalence of underlying medical conditions among Black children,^23,24^ may explain why disparities in rates of severe illness persisted even as overall hospitalization rates decreased.

This study highlights the importance of underlying medical conditions as a possible factor associated with higher COVID-19 hospitalization rates among Black children. In particular, asthma rates, which have been demonstrated to increase risk for severe COVID-19 disease,^7^ are higher among Black children^23,25^ compared with other racial and ethnic groups, a finding confirmed by our study that showed 19.5% of Black children hospitalized for COVID-19 had asthma. In addition, 11.9% of Black children hospitalized for COVID-19 from October 2022 to September 2023 had sickle cell disease, a high prevalence given that sickle cell disease occurs in approximately 0.3% of Black births in the US.^26^ Our study also found that a higher proportion of Black children hospitalized for COVID-19 were in the older pediatric age groups (5–11 and 12–17 years) compared with other hospitalized children. Older age at hospitalization suggests inequitable access to care and a higher incidence of chronic conditions, further highlighting disparities among different racial and ethnic groups. Increasing vaccination rates and access to early treatment for all eligible children, particularly those with underlying medical conditions that increase risk of COVID-19 hospitalization, may help reduce persistent racial and ethnic disparities in COVID-19 hospitalization.

The persistent racial and ethnic disparities in ICU admission and hospitalization rates identified in COVID-NET are concerning, given the increasing availability of COVID-19 vaccines and treatments over the study period, beginning with the availability of the COVID-19 vaccine for all children aged 6 months or older in June 2022. Vaccine coverage was likely associated with racial and ethnic disparities in hospitalizations. Studies have documented that COVID-19 vaccine uptake has been low among children of all races and ethnicities,^27^ and 1 study found that intention to vaccinate children was lowest among Black and Hispanic caregivers compared with White and Asian or Pacific Islander caregivers.^28^ In our analysis, among children hospitalized for COVID-19 from October 2022 to September 2023, almost all vaccine-eligible children, including more than 96% of both Black and Hispanic children, had not received the most recent recommended dose, highlighting an opportunity to prevent severe disease in these groups if vaccination coverage can be increased.

In addition to differences in underlying medical conditions^24,29,30^ and vaccine uptake^28^ that may be associated with racial and ethnic disparities in pediatric COVID-19 hospitalizations, disparities may also be associated with such variables as systemic social inequities^5,31,32^ and decreased access to health care.^33^ In our study, a higher proportion of hospitalized Black and Hispanic children had public insurance compared with their White and Asian or Pacific Islander peers, who were more likely to have private insurance. Although this analysis was not designed to examine reasons for observed disparities by race and ethnicity, previous studies have highlighted that COVID-19–related disparities may be attenuated by addressing factors associated with socioeconomic status, increased access to health care, and early antiviral treatment among eligible patients.^31,32,34,35^

### Limitations

This analysis has several limitations. COVID-NET patients were identified by clinician-driven testing and may not capture all COVID-19–associated hospitalizations, as testing practices have changed over time and some hospitalized patients with COVID-19 may not have been tested. Hospitalization rates may be underestimated for all races, as those categorized as Hispanic could be of any race and those who identified with Hispanic ethnicity and another racial category would be categorized as Hispanic only. Limited data were available for patients who identified as more than 1 race and for patients identified as American Indian or Alaska Native, which limited analyses for these groups, although additional future analyses are planned for these groups. Race and ethnicity data are obtained from medical record review and subject to misclassification; for cases with unknown race or ethnicity, these data may not be missing at random,^30^ although unknown race was limited to a small proportion of cases (5%), and thus excluding them was unlikely to affect results. In addition, this analysis captures differences in population-based hospitalization rates by race and ethnicity but, besides insurance status, did not have additional data available related to other aspects of social determinants of health, which are important to consider when addressing the causes of health disparities by race and ethnicity. Differential access to health care potentially affects hospitalization rates by underestimating COVID-19 hospitalizations for populations with less access to care or different thresholds for hospitalization. Less access to care may in turn result in presentation of patients with more severe disease at the time of hospitalization and may reflect an increased proportion of those who require a higher level of care. Last, the catchment area for COVID-NET cases covers approximately 10% of the US population and may not be representative of the general US population, although COVID-NET trends are similar to those observed nationally.^36^

## Conclusions

In this cross-sectional study, population-based surveillance identified more than 13 000 pediatric COVID-19–associated hospitalizations from March 2020 to September 2023. Black and Hispanic children experienced hospitalizations for severe COVID-19 disease at higher rates than their White and Asian or Pacific Islander peers. Although hospitalization and ICU admission rates decreased overall since the start of the COVID-19 pandemic and disparities have narrowed, racial and ethnic disparities persist. These disparities may be associated in part with the higher proportion of Black children with underlying medical conditions, including asthma and sickle cell disease, and may be exacerbated by disparities in health care access and other social determinants of health. Black and Hispanic children and other disproportionately affected groups may benefit from additional public health interventions, including equitable access to health care, vaccines, and treatment, as well as interventions from policymakers, clinicians, and public health practitioners that strive to narrow gaps by focusing on populations experiencing disproportionate effects and multiple inequities that intersect with race and ethnicity.

## Supplementary Material

Supplement 1 (eFigure 1-eFigure 2c & eTable) **eFigure 1.** COVID-NET Pediatric Hospitalizations Flow Diagram
**eFigure 2A.** Cumulative Pediatric COVID-19 Hospitalization Rates by Race and Ethnicity, and Respiratory Virus Surveillance Period March 2020-September 2023, Children Aged 0–4 Years
**eFigure 2B.** Cumulative Pediatric COVID-19 Hospitalization Rates by Race and Ethnicity, and Respiratory Virus Surveillance Period: March 2020-September 2023, Children Aged 5–11 Years
**eFigure 2C.** Cumulative Pediatric COVID-19 Hospitalization Rates by Race and Ethnicity, and Respiratory Virus Surveillance Period March 2020-September 2023, Children Aged 12–17 Years
**eTable.** Characteristics of Excluded Pediatric Cases Admitted for COVID-19 October 2022-September 2023 by Race and Ethnicity

Supplement 2 (Data Sharing Statement) Data Sharing Statement

Supplemental content

Author affiliations and article information are listed at the end of this article.

## Figures and Tables

**Table 1.** Characteristics of Pediatric COVID-19–Associated Hospitalizations, 2020–2023^a^

	Hospitalizations, No. (%)				
Characteristic	Total (N = 13 555)	2020 (n = 785)	2020–2021 (%) (n = 3192)	2021–2022 (n = 6667)	2022–2023 (%) (n = 2911)
Age, median (IQR), y	3.3 (0.6–12.5)	8.0 (0.9–14.7)	9.1 (1.0–14.5)	2.6 (0.6–11.7)	1.0 (0.3–7.8)
Age group					
≤6 mo	2939 (21.7)	154 (19.6)	488 (15.3)	1465 (22.0)	832 (28.6)
6 mo-4 y	4157 (30.7)	167 (21.3)	677 (21.2)	2214 (33.2)	1099 (37.8)
5–11 y	2427 (17.9)	132 (16.8)	661 (20.7)	1199 (18.0)	435 (14.9)
12–17 y	4032 (29.7)	332 (42.3)	1366 (42.8)	1789 (26.8)	545 (18.7)
Sex					
Male	7110 (52.5)	412 (52.5)	1561 (48.9)	3546 (53.2)	1591 (54.7)
Female	6445 (47.5)	373 (47.5)	1631 (51.1)	3121 (46.8)	1320 (45.3)
Race and ethnicity^b^					
Asian or Pacific Islander	780 (5.8)	39 (5.0)	157 (4.9)	384 (5.8)	200 (6.9)
Black	3837 (28.3)	259 (33.0)	1110 (34.8)	1810 (27.1)	658 (22.6)
Hispanic	4131 (30.5)	376 (47.9)	964 (30.2)	1841 (27.6)	950 (32.6)
White	4807 (35.5)	111 (14.1)	961 (30.1)	2632 (39.5)	1103 (37.9)

a Each surveillance period spans from October of the earliest year to September of the following year, with exception of 2020, when surveillance began in March 2020 and continued through September 2020.

b Race and ethnicity were categorized as follows: non-Hispanic Asian or Pacific Islander, non-Hispanic Black, Hispanic, and non-Hispanic White. If ethnicity was unknown, non-Hispanic ethnicity was assumed.

**Table 2.** Hospitalization and ICU Admission Rates and Rate Ratios of Pediatric Cases Admitted for COVID-19 by Race, Ethnicity, and Age Group, October 2022 to September 2023^a,b,c^

	Asian or Pacific Islander		Black		Hispanic		White	
Outcome	Rate per 100 000 (95% Cl)	Rate ratio (95% Cl)	Rate per 100 000 (95% Cl)	Rate ratio (95% Cl)	Rate per 100 000 (95% Cl)	Rate ratio (95% Cl)	Rate per 100 000 (95% Cl)	Rate ratio (95% Cl)
**Overall**								
Hospitalization rate	35.7 (30.8–40.7)	1 [Reference]	51.9 (47.9–55.8)	1.45 (1.24–1.70)	66.2 (62.0–70.4)	1.85 (1.59–2.16)	38.6 (36.3–40.9)	1.08 (0.93–1.26)
ICU admission rate	5.8 (3.8–7.8)	1 [Reference]	10.9 (9.1–12.8)	1.88 (1.28–2.75)	12.4 (10.6–14.3)	2.13 (1.47–3.10)	6.8 (5.8–7.7)	1.16 (0.80–1.69)
**Aged ≤4 y**								
Hospitalization rate	96.1 (80.4–111.8)	1 [Reference]	126.8 (114.6–139.1)	1.32 (1.09–1.60)	169.2 (156.1–182.2)	1.76 (1.47–2.11)	102.8 (95.3–110.3)	1.07 (0.89–1.28)
ICU admission rate	13.3 (7.4–19.1)	1 [Reference]	29.8 (23.9–35.7)	2.25 (1.39–3.64)	35.1 (29.2–41.0)	2.65 (1.65–4.24)	19.7 (16.5–23.0)	1.49 (0.93–2.38)
**Aged 5–11 y**								
Hospitalization rate	12.6 (8.0–17.3)	1 [Reference]	21.5 (17.4–25.6)	1.70 (1.12–2.59)	25.7 (21.5–30.0)	2.04 (1.36–3.06)	14.7 (12.4–16.9)	1.16 (0.78–1.73)
ICU admission rate	1.3 (0.0–2.9)	1 [Reference]	4.7 (2.8–6.6)	3.50 (1.04–11.72)	2.6 (1.3–4.0)	1.95 (0.56–6.83)	2.5 (1.6–3.4)	1.88 (0.57–6.21)
**Aged 12–17 y**								
Hospitalization rate	14.9 (9.4–20.4)	1 [Reference]	30.8 (25.7–35.9)	2.07 (1.38–3.10)	31.8 (26.9–36.7)	2.14 (1.43–3.19)	20.5 (17.8–23.3)	1.38 (0.93–2.05)
ICU admission rate	5.2 (1.9–8.5)	1 [Reference]	4.1 (2.3–6.0)	0.80 (0.37–1.72)	5.8 (3.7–7.9)	1.12 (0.54–2.30)	2.6 (1.6–3.6)	0.50 (0.24–1.03)

Abbreviations: COVID-NET, COVID-19 Hospitalization Surveillance Network; ICU, intensive care unit.

a Race and ethnicity were categorized as follows: non-Hispanic Asian or Pacific Islander, non-Hispanic Black, Hispanic, and non-Hispanic White. If ethnicity was unknown, non-Hispanic ethnicity was assumed.

b Cumulative hospitalization rates per 100 000 population were calculated using all hospitalized pediatric cases in COVID-NET with known race and ethnicity for the numerator and National Center for Health Statistics vintage 2020 unbridged-race population estimates for the denominator.

c From October 2022 to September 2023, some sites completed medical record abstractions for all pediatric COVID-NET cases while the remainder completed a random sample. Random numbers (1–100) were generated and assigned to each case to produce a random sample for medical record abstraction stratified by site, month, and age group. Intensive care unit admission status was calculated based on medical record review for sampled hospitalized patients with known race and ethnicity.

**Table 3.** Characteristics of Pediatric Cases Admitted for COVID-19 by Race and Ethnicity, October 2022 to September 2023^a,b,c^

	Total (N = 2169)		Asian or Pacific Islander (n = 152)		Black (n = 490)		Hispanic (n = 726)		White (n = 801)		P value determined by χ^2^ test
Characteristic	No.	% (95% Cl)	No.	% (95% Cl)	No.	% (95% Cl)	No.	% (95% Cl)	No.	% (95% Cl)	
Age, median (IQR), y	2169	0.9 (0.3–4.2)	152	1.0 (0.3–3.7)	490	1.0 (0.4–6.3)	726	0.9 (0.2–3.4)	801	0.9 (0.3–3.8)	<.001
Age group											
≤6 mo	671	31.2 (29.2–33.2)	41	27.2 (20.3–35.0)	124	25.5 (21.7–29.6)	247	34.1 (30.7–37.7)	259	32.7 (29.4–36.1)	.04
6 mo-4 y	940	43.3 (41.2–45.5)	74	48.6 (40.5–56.9)	215	43.9 (39.4–48.4)	307	42.5 (38.9–46.2)	344	42.8 (39.3–46.3)	
5–11 y	308	14.1 (12.7–15.6)	23	15.2 (9.9–21.9)	82	16.6 (13.5–20.2)	97	13.2 (10.9–15.9)	106	13.1 (10.9–15.7)	
12–17 y	250	11.4 (10.1–12.8)	14	9.0 (5.0–14.7)	69	13.9 (11.0–17.3)	75	10.1 (8.0–12.6)	92	11.4 (9.3–13.8)	
Sex											
Male	1237	56.9 (54.8–59.0)	86	56.2 (47.9–64.2)	281	57.3 (52.8–61.7)	411	56.5 (52.7–60.1)	459	57.3 (53.8–60.7)	.98
Female	932	43.1 (41.0–45.2)	66	43.8 (35.8–52.1)	209	42.7 (38.3–47.2)	315	43.5 (39.9–47.3)	342	42.7 (39.3–46.2)	
Insurance											
Private	1048	49.7 (47.6–51.9)	84	57.6 (49.1–65.7)	191	40.0 (35.6–44.6)	186	26.4 (23.2–29.9)	587	75.5 (72.4–78.5)	<.001
Medicaid	993	47.3 (45.2–49.5)	60	41.1 (33.0–49.5)	275	58.0 (53.5–62.5)	482	68.5 (64.9–71.9)	176	22.5 (19.6–25.6)	
Uninsured	62	2.9 (2.3–3.8)	<10	1.3 (0.2–4.8)	<10	1.9 (0.9–3.6)	36	5.1 (3.6–7.0)	15	1.9 (1.1–3.2)	
Any underlying medical condition	1046	48.0 (45.9–50.1)	65	42.3 (34.4–50.6)	299	61.0 (56.6–65.4)	325	44.6 (40.9–48.3)	357	44.3 (40.8–47.8)	<.001
No, of underlying medical conditions											
0	1100	50.9 (48.8–53.1)	83	55.0 (46.8–63.1)	189	38.6 (34.2–43.0)	394	54.5 (50.8–58.1)	434	54.4 (50.9–57.9)	<.001
1	552	25.4 (23.6–27.3)	35	22.9 (16.4–30.4)	150	30.8 (26.7–35.1)	170	23.3 (20.3–26.6)	197	24.5 (21.5–27.6)	
≥2	517	23.7 (21.9–25.5)	34	22.1 (15.8–29.6)	151	30.7 (26.6–35.0)	162	22.2 (19.2–25.4)	170	21.1 (18.3–24.1)	
Obesity^d^	NS	17.8 (15.3–20.5)	<10	14.3 (6.8–25.4)	42	19.3 (14.3–25.2)	53	19.5 (14.9–24.7)	48	15.9 (11.9–20.5)	.54
Diabetes	NS	2.5 (1.9–3.2)	<10	NA	19	3.8 (2.3–5.9)	12	1.6 (0.8–2.8)	23	2.9 (1.8–4.3)	NA
Asthma	295	13.5 (12.1–15.0)	17	11.1 (6.6–17.2)	96	19.5 (16.1–23.3)	89	12.2 (9.9–14.8)	93	11.5 (9.4–13.9)	<.001
Cardiovascular disease	193	8.9 (7.7–10.1)	15	9.8 (5.6–15.7)	44	8.9 (6.6–11.8)	62	8.5 (6.6–10.8)	72	8.9 (7.0–11.1)	.96
Congenital cardiovascular disease	131	6.0 (5.1–7.1)	12	7.9 (4.1–13.3)	25	5.1 (3.3–7.5)	46	6.3 (4.7–8.4)	48	5.9 (4.4–7.8)	.63
Neurologic disorders	331	15.2 (13.7–16.7)	26	16.8 (11.3–23.7)	81	16.4 (13.3–20.0)	109	15.0 (12.5–17.8)	115	14.3 (11.9–16.9)	.69
Kidney disorders	23	1.0 (0.7–1.6)	<10	1.3 (0.2–4.6)	<10	1.6 (0.7–3.2)	<10	0.9 (0.4–1.9)	<10	0.7 (0.3–1.6)	.48
Immunosuppressive conditions	NS	6.0 (5.1–7.1)	<10	3.8 (1.4–8.2)	26	5.3 (3.5–7.6)	39	5.3 (3.8–7.2)	61	7.5 (5.8–9.6)	.12
Blood disorders, including sickle cell disease	NS	5.3 (4.4–6.3)	<10	2.6 (0.7–6.5)	65	13.5 (10.5–16.9)	20	2.7 (1.7–4.2)	25	3.1 (2.0–4.5)	<.001
Sickle cell disease	59	2.8 (2.1–3.5)	<10	0.7 (0.02–3.6)	57	11.9 (9.1–15.1)	<10	NA	<10	0.1 (0.0–0.7)	NA
Rheumatologic or autoimmune disease	<10	0.3 (0.1–0.6)	<10	NA	<10	0.2 (0.01–1.1)	<10	0.4 (0.1–1.2)	<10	0.2 (0.03–0.9)	NA
Clinical outcomes and interventions											
Length of stay, median (IQR), d	2168	1.7 (0.8–3.4)	152	1.9 (1.0–3.7)	490	1.9 (0.9–3.7)	725	1.7 (0.8–3.5)	801	1.6 (0.7–3.1)	.39
ICU	543	24.9 (23.1–26.8)	33	21.5 (15.2–28.8)	139	28.3 (24.3–32.5)	177	24.2 (21.1–27.5)	194	24.1 (21.2–27.2)	.22
Mechanical ventilation	135	6.2 (5.2–7.3)	13	8.4 (4.5–14.0)	38	7.7 (5.5–10.4)	46	6.4 (4.7–8.4)	38	4.7 (3.4–6.4)	.10
Death	11	0.5 (0.2–0.9)	<10	0.6 (0.02–3.6)	<10	0.4 (0.05–1.5)	<10	0.5 (0.1–1.4)	<10	0.5 (0.1–1.3)	.98
Vaccination status^e^											
No record of COVID-19 vaccination	1260	84.5 (82.6–86.3)	93	84.6 (76.5–90.8)	330	90.4 (86.9–93.2)	418	87.4 (84.1–90.2)	419	77.9 (74.2–81.4)	<.001
Received ≥1 dose, but not up to date	160	10.7 (9.2–12.4)	14	12.7 (7.1–20.4)	22	6.0 (3.8–9.0)	44	9.1 (6.7–12.0)	80	14.8 (12.0–18.1)	
Up to date	NS	4.8 (3.8–6.0)	<10	2.7 (0.5–7.7)	13	3.6 (1.9–6.0)	17	3.5 (2.0–5.6)	39	7.2 (5.2–9.8)	

Abbreviations: COVID-NET, COVID-19 Hospitalization Surveillance Network; ICU, intensive care unit; NA, not applicable; NS, not shown.

a Race and ethnicity were categorized as follows: non-Hispanic Asian or Pacific Islander, non-Hispanic Black, Hispanic, and non-Hispanic White. If ethnicity was unknown, non-Hispanic ethnicity was assumed.

b From October 2022 to September 2023, some sites completed medical record abstractions for all pediatric COVID-NET cases, while the remainder completed a random sample. Random numbers (1–100) were generated and assigned to each case to produce a random sample for medical record abstraction stratified by site, month, and age group. Unweighted counts and weighted column percentages are presented for these sampled data that better represent the hospitalized population of the COVID-NET catchment area.

c Cells with observations less than 10 are suppressed for privacy and confidentiality, and total counts are not shown when only a single race or ethnicity had observations less than 10 for a given condition.

d Calculation for obesity is determined by documented medical history or documented measurement of body mass index in the medical record and excludes children younger than 2 years.

e Children aged younger than 6 months are not eligible to receive the COVID-19 vaccine and are excluded from these calculations. COVID-19 vaccination eligibility changed by age group over time during the study period. Vaccine-eligible children and adolescents ages 6 months or older without any evidence of ever having received a COVID-19 vaccination were categorized as having no record of COVID-19 vaccination. Before the 2022–2023 (bivalent) formula doses were recommended, children and adolescents who completed a COVID-19 primary vaccination series 14 days or more before the positive SARS-CoV-2 test associated with their hospitalization were categorized as up to date. After the 2022–2023 booster doses were recommended, only those who completed the COVID-19 primary vaccination series and subsequently received a recommended booster dose 14 days or more before testing positive for SARS-CoV-2 were categorized as up to date. Children and adolescents who began, but did not complete, a COVID-19 primary vaccination series, or who completed a primary series but did not receive a recommended booster dose 14 days or more before testing positive for SARS-CoV-2 were categorized as having received at least 1 dose of COVID-19 vaccine but were not up to date.

## Data Availability

See Supplement 2.

## Key Points

### Question

Were there racial and ethnic disparities among pediatric COVID-19–associated hospitalizations?

### Findings

In this cross-sectional study, population-based surveillance identified 13 555 pediatric COVID-19–associated hospitalizations from March 2020 to September 2023. Non-Hispanic Black and Hispanic children had the highest hospitalization and intensive care unit admission rates and were hospitalized at more than twice the rate of non-Hispanic Asian or Pacific Islander children, the group with the lowest hospitalization rates; statistically significant disparities persisted throughout the study period.

### Meaning

This study suggests that non-Hispanic Black and Hispanic children were disproportionately affected by severe COVID-19, highlighting increased need for equitable access and use of COVID-19 therapeutics and vaccines.

## References

[1] Garg S, Patel K, Pham H, Clinical trends among US adults hospitalized with COVID-19, March to December 2020: a cross-sectional study. Ann Intern Med. 2021;174(10):1409–1419. doi:10.7326/M21-1991 34370517 PMC8381761

[2] Ko JY, Pham H, Anglin O, ; COVID-NET Surveillance Team. Vaccination status and trends in adult coronavirus disease 2019–associated hospitalizations by race and ethnicity: March 2020-August 2022. Clin Infect Dis. 2023;77(6):827–838. doi:10.1093/cid/ciad266 37132204 PMC11019819

[3] Delahoy MJ, Ujamaa D, Whitaker M, ; COVID-NET Surveillance Team; COVID-NET Surveillance Team. Hospitalizations associated with COVID-19 among children and adolescents—COVID-NET, 14 states, March 1, 2020–August 14, 2021. MMWR Morb Mortal Wkly Rep. 2021;70(36):1255–1260. doi:10.15585/mmwr.mm7036e2 34499627 PMC8437052

[4] Acosta AM, Garg S, Pham H, Racial and ethnic disparities in rates of COVID-19–associated hospitalization, intensive care unit admission, and in-hospital death in the United States from March 2020 to February 2021. JAMA Netw Open. 2021;4(10):e2130479. doi:10.1001/jamanetworkopen.2021.30479 34673962 PMC8531997

[5] Khanijahani A, Iezadi S, Gholipour K, Azami-Aghdash S, Naghibi D. A systematic review of racial/ethnic and socioeconomic disparities in COVID-19. Int J Equity Health. 2021;20(1):248. doi:10.1186/s12939-021-01582-4 34819081 PMC8611382

[6] Magesh S, John D, Li WT, Disparities in COVID-19 outcomes by race, ethnicity, and socioeconomic status: a systematic review and meta-analysis. JAMA Netw Open. 2021;4(11):e2134147. doi:10.1001/jamanetworkopen.2021.34147 34762110 PMC8586903

[7] Woodruff RC, Campbell AP, Taylor CA, Risk factors for severe COVID-19 in children. Pediatrics. 2022;149(1):e2021053418. doi:10.1542/peds.2021-053418 34935038 PMC9213563

[8] Tai DBG, Shah A, Doubeni CA, Sia IG, Wieland ML. The disproportionate impact of COVID-19 on racial and ethnic minorities in the United States. Clin Infect Dis. 2021;72(4):703–706. doi:10.1093/cid/ciaa815 32562416 PMC7337626

[9] Marks KJ, Whitaker M, Agathis NT, ; COVID-NET Surveillance Team. Hospitalization of infants and children aged 0–4 years with laboratory-confirmed COVID-19—COVID-NET, 14 states, March 2020–February 2022. MMWR Morb Mortal Wkly Rep. 2022;71(11):429–436. doi:10.15585/mmwr.mm7111e2 35298458 PMC8942304

[10] U.S. Census populations with bridged race categories. National Center for Health Statistics, Centers for Disease Control and Prevention. Accessed August 4, 2021. https://www.cdc.gov/nchs/nvss/bridged_race.htm

[11] O’Halloran A, Whitaker M, Patel K, Developing a sampling methodology for timely reporting of population-based COVID-19–associated hospitalization surveillance in the United States, COVID-NET 2020–2021. Influenza Other Respir Viruses. 2023;17(1):e13089. doi:10.1111/irv.13089 36625234 PMC9835436

[12] Interim clinical considerations for use of COVID-19 vaccines in the United States. Centers for Disease Control and Prevention. 2025. Accessed November 27, 2024. https://www.cdc.gov/covid/hcp/vaccine-considerations/index.html

[13] Taylor CA, Whitaker M, Patton ME, Trends in COVID-19–attributable hospitalizations among adults with laboratory-confirmed SARS-CoV-2—COVID-NET, June 2020 to September 2023. Influenza Other Respir Viruses. 2024;18(11):e70021. doi:10.1111/irv.70021 39496579 PMC11534501

[14] Yehia BR, Winegar A, Fogel R, Association of race with mortality among patients hospitalized with coronavirus disease 2019 (COVID-19) at 92 US hospitals. JAMA Netw Open. 2020;3(8):e2018039. doi:10.1001/jamanetworkopen.2020.18039 32809033 PMC7435340

[15] Marks KJ, Whitaker M, Anglin O, ; COVID-NET Surveillance Team. Hospitalizations of children and adolescents with laboratory-confirmed COVID-19—COVID-NET, 14 states, July 2021–January 2022. MMWR Morb Mortal Wkly Rep. 2022;71(7):271–278. doi:10.15585/mmwr.mm7107e4 35176003 PMC8853476

[16] Phelan JC, Link BG, Tehranifar P. Social conditions as fundamental causes of health inequalities: theory, evidence, and policy implications. J Health Soc Behav. 2010;51(1)(suppl):S28–S40. doi:10.1177/0022146510383498 20943581

[17] Gaskin DJ, Thorpe RJ Jr, McGinty EE, Disparities in diabetes: the nexus of race, poverty, and place. Am J Public Health. 2014;104(11):2147–2155. doi:10.2105/AJPH.2013.301420 24228660 PMC4021012

[18] Millett GA, Jones AT, Benkeser D, Assessing differential impacts of COVID-19 on Black communities. Ann Epidemiol. 2020;47:37–44. doi:10.1016/j.annepidem.2020.05.003 32419766 PMC7224670

[19] Scannell CA, Oronce CIA, Tsugawa Y. Association between county-level racial and ethnic characteristics and COVID-19 cases and deaths in the USA. J Gen Intern Med. 2020;35(10):3126–3128. doi:10.1007/s11606-020-06083-8 32761284 PMC7406133

[20] Jones JM, Opsomer JD, Stone M, Updated US infection- and vaccine-induced SARS-CoV-2 seroprevalence estimates based on blood donations, July 2020-December 2021. JAMA. 2022;328(3):298–301. doi:10.1001/jama.2022.9745 35696249 PMC9194752

[21] Aparicio C, Willis ZI, Nakamura MM, Risk factors for pediatric critical COVID-19: a systematic review and meta-analysis. J Pediatric Infect Dis Soc. 2024;13(7):352–362. doi:10.1093/jpids/piae052 38780125 PMC11519042

[22] Havers FP. COVID-19–associated hospitalizations—COVID-NET, April 2025 update. Presented at: Advisory Committee on Immunization Practices (ACIP) Meeting; April 15, 2025; Atlanta, Georgia. Accessed April 28, 2025. https://www.cdc.gov/acip/downloads/slides-2025-04-15-16/03-Havers-COVID-508.pdf

[23] Kim C, Stierman B, Hales CM, Ogden CL. Race and Hispanic-origin disparities in underlying medical conditions associated with severe COVID-19 illness: US adults, 2015–2018. April 28, 2021. National Center for Health Statistics; Centers for Disease Control and Prevention. Accessed October 7, 2024. https://www.cdc.gov/nchs/data/nhsr/nhsr154-508.pdf

[24] Kompaniyets L, Agathis NT, Nelson JM, Underlying medical conditions associated with severe COVID-19 illness among children. JAMA Netw Open. 2021;4(6):e2111182. doi:10.1001/jamanetworkopen.2021.11182 34097050 PMC8185607

[25] Most recent national asthma data. Centers for Disease Control and Prevention. Accessed July 17, 2024. https://www.cdc.gov/asthma/most_recent_national_asthma_data.htm

[26] Data and statistics on sickle cell disease. Centers for Disease Control and Prevention. Accessed June 28, 2024. https://www.cdc.gov/sickle-cell/data/index.html

[27] COVID-19 vaccination coverage and intent for vaccination, children 6 months through 17 years, United States. Centers for Disease Control and Prevention. Updated January 23, 2024. Accessed October 7, 2024. https://www.cdc.gov/covidvaxview/weekly-dashboard/child-coverage-vaccination.html?CDC_AAref_Val=https://www.cdc.gov/vaccines/imz-managers/coverage/covidvaxview/interactive/children-coverage-vaccination.html

[28] Hart RJ, Baumer-Mouradian S, Bone JN, ; International COVID-19 Parental Attitude Study (COVIPAS) Group. Factors associated with US caregivers’ uptake of pediatric COVID-19 vaccine by race and ethnicity. Vaccine. 2023;41(15):2546–2552. doi:10.1016/j.vaccine.2023.02.080 36906408 PMC9986131

[29] Moore JT, Pilkington W, Kumar D. Diseases with health disparities as drivers of COVID-19 outcome. J Cell Mol Med. 2020;24(19):11038–11045. doi:10.1111/jcmm.15599 32816409 PMC7461081

[30] Keppel K, Pamuk E, Lynch J, Methodological issues in measuring health disparities. Vital Health Stat 2. 2005;(141):1–16. PMC3681823 16032956

[31] Goyal MK, Simpson JN, Boyle MD, Racial and/or ethnic and socioeconomic disparities of SARS-CoV-2 infection among children. Pediatrics. 2020;146(4):e2020009951. doi:10.1542/peds.2020-009951 32759379

[32] Vicetti Miguel CP, Dasgupta-Tsinikas S, Lamb GS, Olarte L, Santos RP. Race, ethnicity, and health disparities in US children with COVID-19: a review of the evidence and recommendations for the future. J Pediatric Infect Dis Soc. 2022;11(suppl 4):S132–S140. doi:10.1093/jpids/piac099 36063366 PMC9494369

[33] Kim C, Yee R, Bhatkoti R, COVID-19 vaccine provider access and vaccination coverage among children aged 5–11 years—United States, November 2021–January 2022. MMWR Morb Mortal Wkly Rep. 2022;71(10):378–383. doi:10.15585/mmwr.mm7110a4 35271559 PMC8911999

[34] Holmes L Jr, Wu C, Hinson R, Black-White risk differentials in pediatric COVID-19 hospitalization and intensive care unit admissions in the USA. J Racial Ethn Health Disparities. 2023;10(3):1187–1193. doi:10.1007/s40615-022-01305-7 35604543 PMC9126624

[35] Havers FP, Pham H, Taylor CA, COVID-19–associated hospitalizations among vaccinated and unvaccinated adults 18 years or older in 13 US States, January 2021 to April 2022. JAMA Intern Med. 2022;182(10):1071–1081. doi:10.1001/jamainternmed.2022.4299 36074486 PMC9459904

[36] Devine O, Pham H, Gunnels B, Extrapolating sentinel surveillance information to estimate national COVID hospital admission rates: a bayesian modeling approach. Influenza Other Respir Viruses. 2024;18(10):e70026. doi:10.1111/irv.70026 39440677 PMC11497105

