# Supplementary material for: Pediatric COVID-19 Hospitalization Trends by Race and Ethnicity, 2020–2023
Source: JAMA Netw Open. Author manuscript; Available in PMC 2026 Jan 27. (PMC12842959; doi:10.1001/jamanetworkopen.2025.21009)
Supplement: Supplement 1 (eFigure 1-eFigure 2c & eTable) — eFigure 1. COVID-NET Pediatric Hospitalizations Flow Diagram eFigure 2A. Cumulative Pediatric COVID-19 Hospitalization Rates by Race and Ethnicity, and Respiratory Virus Surveillance Period March 2020-September 2023, Children Aged 0–4 Years eFigure 2B. Cumulative Pediatric COVID-19 Hospitalization Rates by Race and Ethnicity, and Respiratory Virus Surveillance Period: March 2020-September 2023, Children Aged 5–11 Years eFigure 2C. Cumulative Pediatric COVID-19 Hospitalization Rates by Race and Ethnicity, and Respiratory Virus Surveillance Period March 2020-September 2023, Children Aged 12–17 Years eTable. Characteristics of Excluded Pediatric Cases Admitted for COVID-19 October 2022-September 2023 by Race and Ethnicity [file NIHMS2134914-supplement-Supplement_1__eFigure_1-eFigure_2c___eTable_.pdf]

Supplementary Online Content

Anglin O, Patel K, Daily Kirley P, et al. Pediatric COVID-19 hospitalization trends by race and ethnicity, 2020-2023. *JAMA Netw Open.* 2025;8(7):e2521009. doi:10.1001/jamanetworkopen.2025.21009

eFigure 1. COVID-NET Pediatric Hospitalizations Flow Diagram

eFigure 2A. Cumulative Pediatric COVID-19 Hospitalization Rates by Race and Ethnicity, and Respiratory Virus Surveillance Period March 2020-September 2023, Children Aged 0-4 Years

eFigure 2B. Cumulative Pediatric COVID-19 Hospitalization Rates by Race and Ethnicity, and Respiratory Virus Surveillance Period: March 2020-September 2023, Children Aged 5-11 Years

eFigure 2C. Cumulative Pediatric COVID-19 Hospitalization Rates by Race and Ethnicity, and Respiratory Virus Surveillance Period March 2020-September 2023, Children Aged 12-17 Years

eTable. Characteristics of Excluded Pediatric Cases Admitted for COVID-19 October 2022-September 2023 by Race and Ethnicity

This supplementary material has been provided by the authors to give readers additional information about their work.

eFigure 1. COVID-NET Pediatric Hospitalizations Flow Diagram[a,b,c]

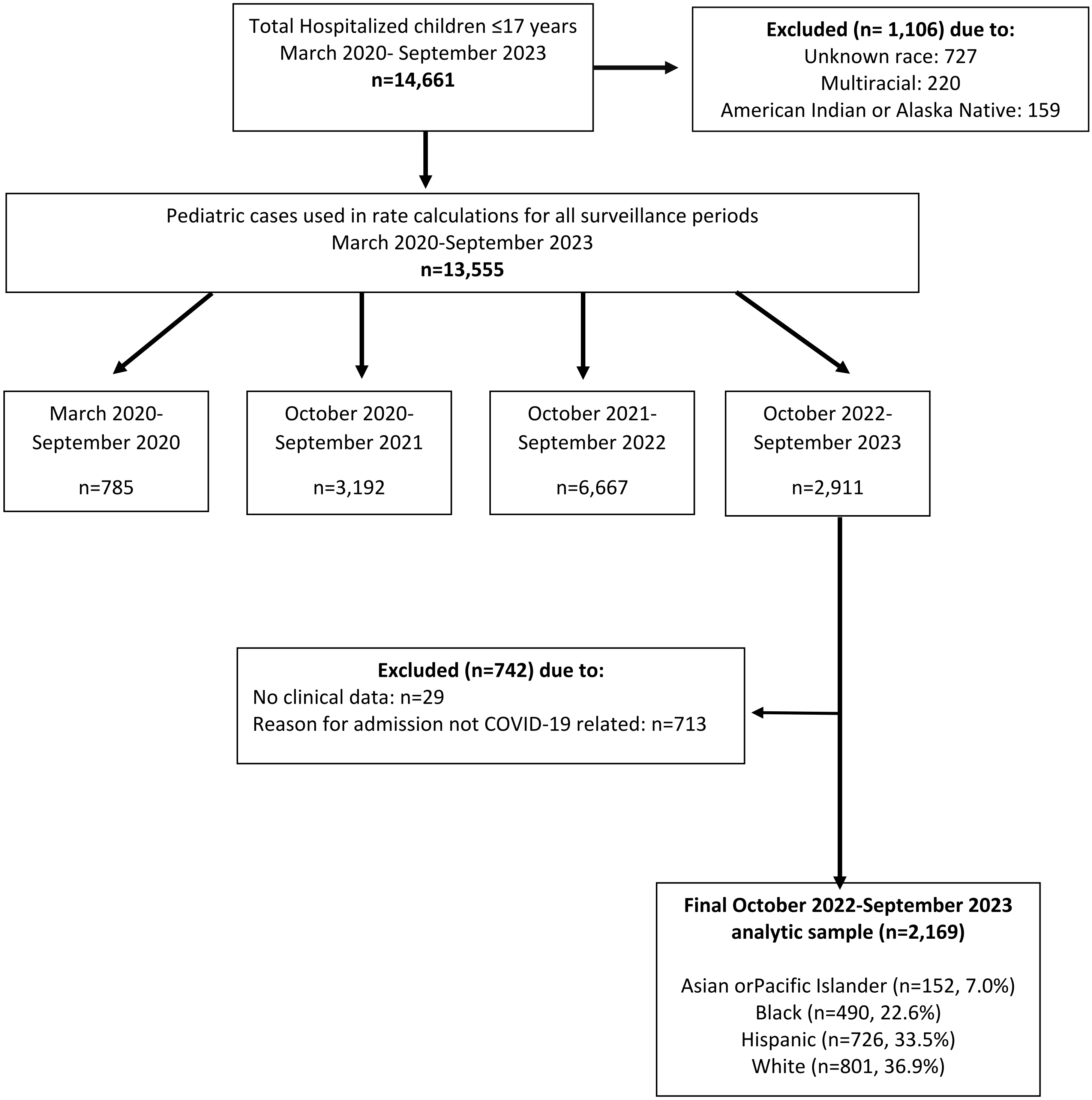

[a] Abbreviations: COVID-NET, COVID-19 Hospitalization Network

[b] Race and ethnicity were categorized as follows: Non-Hispanic Asian or Pacific Islander, non-Hispanic Black, Hispanic, and non-Hispanic White. If ethnicity was unknown, non-Hispanic ethnicity was assumed

[c] During October 2022-September 2023 some sites completed medical chart abstractions for all pediatric COVID-NET cases while the remainder completed a random sample. Random numbers (1-100) were generated and assigned to each case to produce a random sample for medical record abstraction stratified by site, month, and age group.

© 2025 Anglin O et al. *JAMA Network Open.*

eFigure 2A. Cumulative Pediatric COVID-19 Hospitalization Rates by Race and Ethnicity, and Respiratory Virus Surveillance Period March 2020-September 2023, Children Aged 0-4 Years[b]

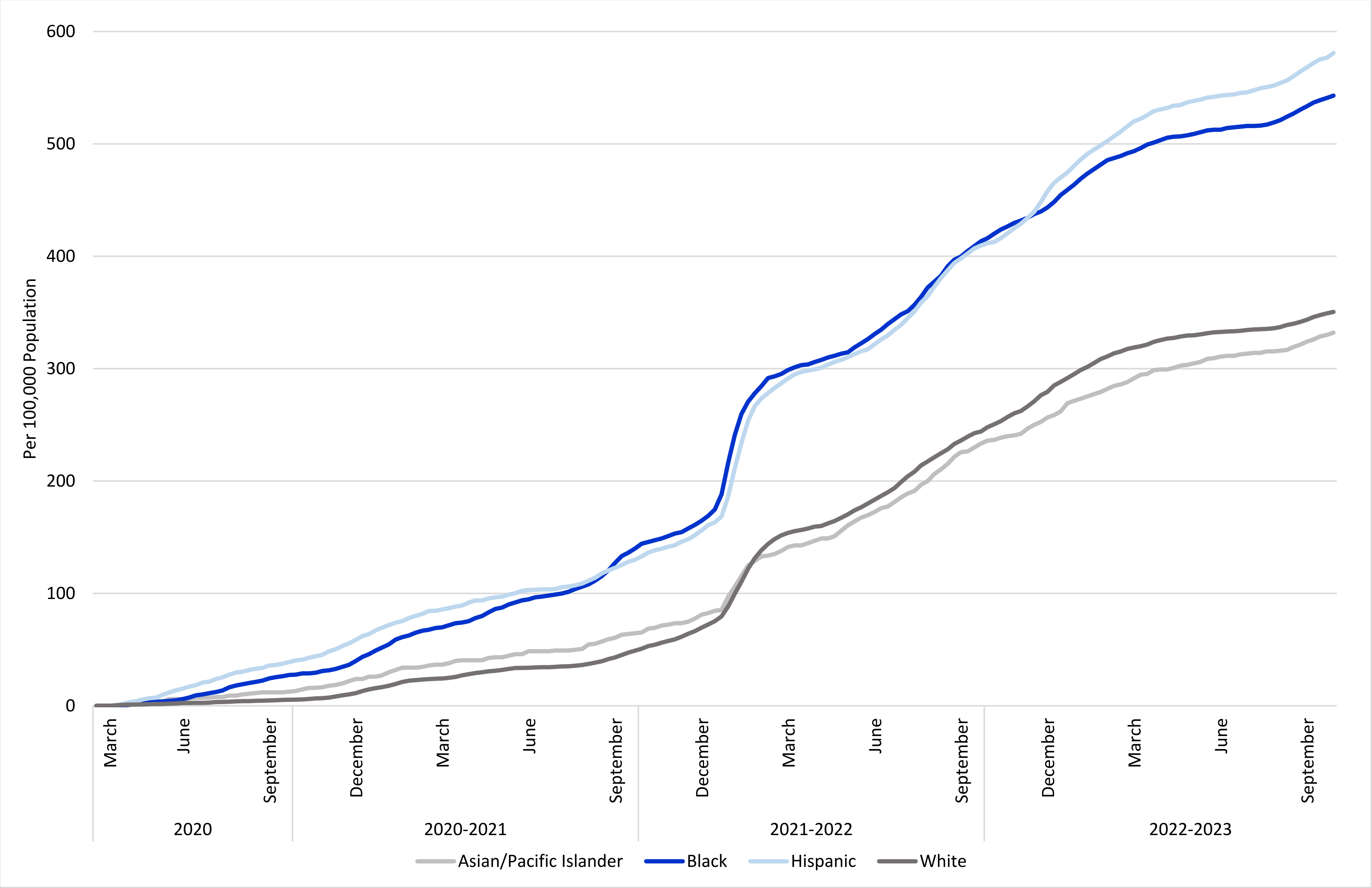

© 2025 Anglin O et al. *JAMA Network Open.*

eFigure 2B. Cumulative Pediatric COVID-19 Hospitalization Rates by Race and Ethnicity, and Respiratory Virus Surveillance Period: March 2020-September 2023, Children Aged 5-11 Years[d]

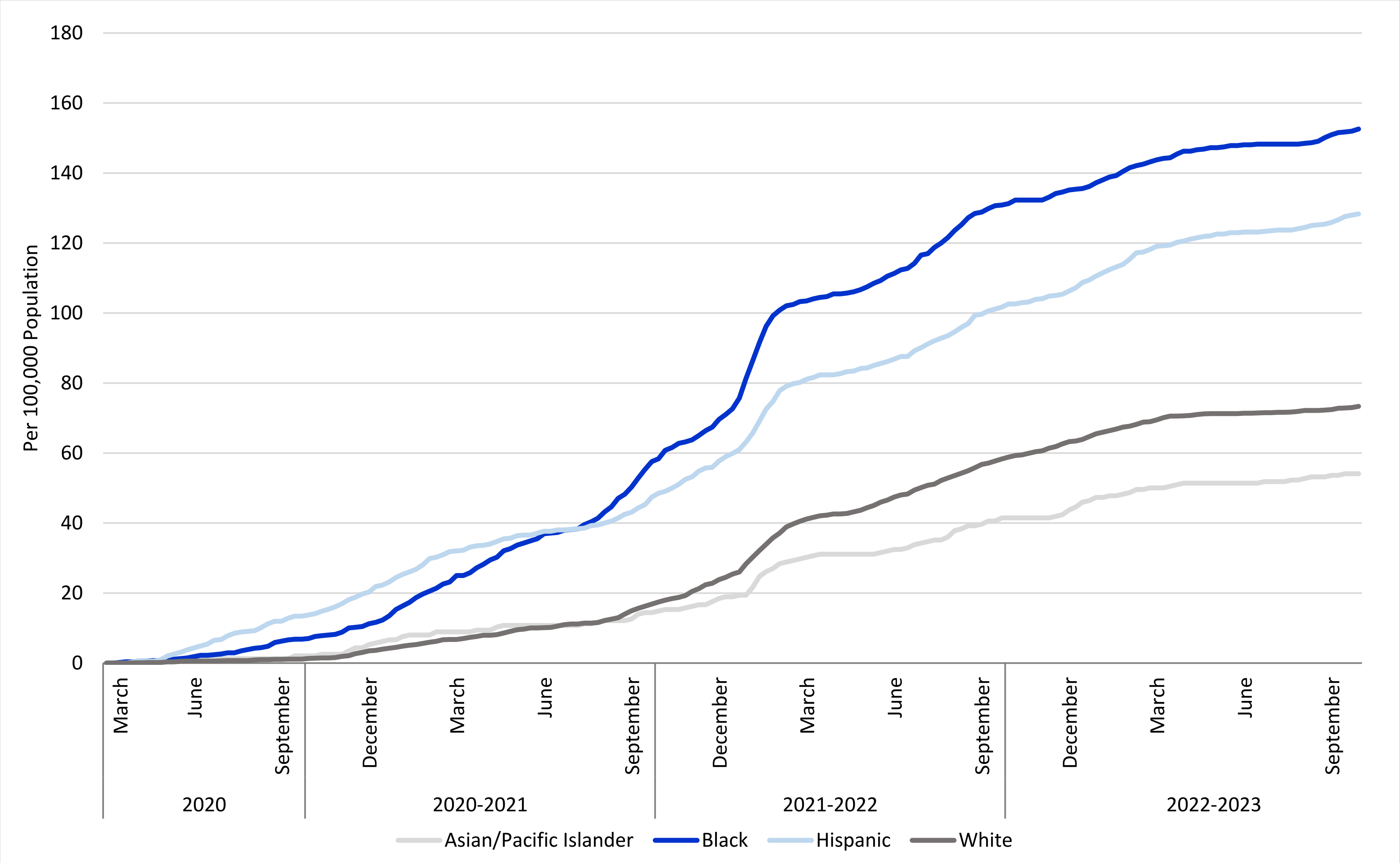

[d] Race and ethnicity were categorized as follows: Non-Hispanic Asian or Pacific Islander, non-Hispanic Black, Hispanic, and non-Hispanic White. If ethnicity was unknown, non-Hispanic ethnicity was assumed.

© 2025 Anglin O et al. *JAMA Network Open.*

eFigure 2C. Cumulative Pediatric COVID-19 Hospitalization Rates by Race and Ethnicity, and Respiratory Virus Surveillance Period March 2020-September 2023, Children Aged 12-17 Years[e]

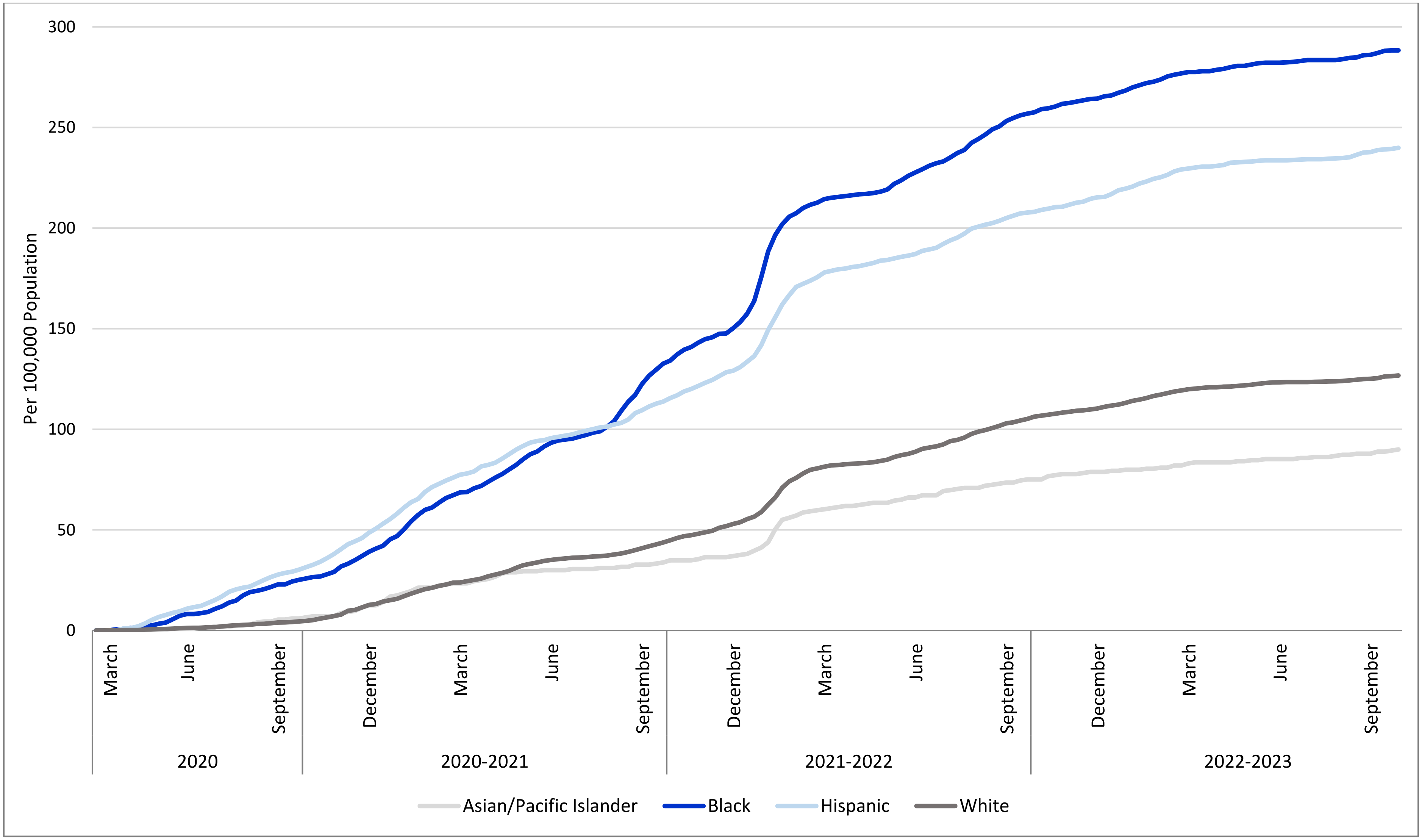

---

[e] Race and ethnicity were categorized as follows: Non-Hispanic Asian or Pacific Islander, non-Hispanic Black, Hispanic, and non-Hispanic White. If ethnicity was unknown, non-Hispanic ethnicity was assumed.

© 2025 Anglin O et al. *JAMA Network Open.*

eTable. Characteristics of Excluded Pediatric Cases Admitted for COVID-19 October 2022-September 2023 by Race and Ethnicity[f,g,h,i]

		Total		American Indian or Alaska Native (AI/AN)		Multiracial		Unknown	
		N	% (95% CI)	N	% (95% CI)	N	% (95% CI)	N	% (95% CI)
**Total**		192		39		59		94	
**Median age (years)**	**Median (IQR)**	192	0.8 (0.24-3.56)	39	0.9 (0.31-5.06)	59	1.0 (0.41-5.71)	94	0.6 (0.16-2.09)
**Age group**	**0-<6 months**	70	36.9 (30.00-44.21)	13	33.5 (19.19-50.35)	15	25.4 (14.96-38.42)	42	45.3 (35.04-55.96)
	**6 mo-4 years**	75	38.8 (31.83-46.07)	15	38.4 (23.29-55.30)	25	42.7 (29.86-56.21)	35	36.6 (26.86-47.13)
	**5-11 years**	Not shown	15.3 (10.56-21.24)	<10	20.6 (9.32-36.51)	11	18.5 (9.56-30.71)	11	11.3 (5.71-19.51)
	**12-17 years**	17	9.0 (5.31-14.00)	<10	7.6 (1.58-20.76)	<10	13.5 (5.99-24.89)	<10	6.8 (2.55-14.11)
**Sex**	**Male**	106	55.7 (48.33-62.84)	23	59.4 (42.47-74.76)	33	55.9 (42.38-68.82)	50	54.0 (43.44-64.36)
	**Female**	86	44.3 (37.16-51.67)	16	40.6 (25.24-57.53)	26	44.1 (31.18-57.62)	44	46.0 (35.64-56.56)
**Insurance**	**Private**	Not shown	52.9 (45.49-60.30)	<10	11.0 (3.15-25.71)	37	65.1 (51.31-77.24)	58	62.1 (51.39-71.96)
	**Medicaid**	86	46.0 (38.68-53.47)	33	89.0 (74.29-96.85)	20	34.9 (22.76-48.69)	33	35.8 (26.12-46.48)
	**Uninsured**	Not shown	1.1 (0.12-3.78)	<10	NA	<10	NA	<10	2.1 (0.24-7.47)
**Any Condition**		84	44.1 (36.89-51.45)	13	33.5 (19.25-50.41)	31	52.7 (39.29-65.87)	40	43.1 (32.90-53.72)

---

[f] Abbreviations: COVID-NET, COVID-19 Hospitalization Network; ICU, intensive care unit; NA, not applicable

[g] Race and ethnicity were categorized as follows: non-Hispanic American Indian/Alaska Native (AI/AN), non-Hispanic Multiracial (multiracial), and Unknown. If ethnicity was unknown, non-Hispanic ethnicity was assumed. Patients categorized as AI/AN, multiracial, or unknown race were excluded from the main analyses due to small case counts, or no population denominator.

[h] During October 2022-September 2023 some sites completed medical chart abstractions for all pediatric COVID-NET cases while the remainder completed a random sample. Random numbers (1-100) were generated and assigned to each case to produce a random sample for medical record abstraction stratified by site, month, and age group. Unweighted counts and weighted percentages are presented for these sampled data that better represent the hospitalized population of the COVID-NET catchment area.

[i] Cells with observations <10 are suppressed for privacy and confidentiality, and total counts are not shown when totals were ≤3 or only a single race/ethnicity had observations <10 for a given condition.

© 2025 Anglin O et al. *JAMA Network Open.*

		Total		American Indian or Alaska Native (AI/AN)		Multiracial		Unknown	
**Number of underlying medical conditions**	**0**	104	53.9 (46.51-61.11)	24	61.4 (44.48-76.52)	26	43.9 (30.99-57.42)	54	56.9 (46.28-67.10)
	**1**	Not shown	26.0 (19.93-32.80)	<10	18.3 (7.77-33.94)	17	28.7 (17.63-41.90)	26	27.5 (18.78-37.66)
	**2 or more**	Not shown	20.1 (14.62-26.65)	<10	20.3 (9.15-36.23)	16	27.5 (16.64-40.63)	14	15.6 (8.73-24.91)
**Obesity[j]**		15	21.8 (12.70-33.35)	<10	33.0 (11.58-61.26)	<10	19.3 (6.61-39.46)	<10	18.0 (6.17-37.09)
**Diabetes**		Not shown	1.0 (0.11-3.65)	<10	NA	<10	1.6 (0.04-9.00)	<10	1.0 (0.02-5.71)
**Asthma**		20	10.2 (6.34-15.42)	<10	10.1 (2.79-24.02)	<10	15.3 (7.24-27.02)	<10	7.2 (2.89-14.44)
**Cardiovascular disease**		18	9.3 (5.58-14.31)	<10	10.5 (2.98-24.49)	<10	10.1 (3.78-20.75)	<10	8.3 (3.61-15.83)
**Congenital Cardiovascular Disease (CVD)**		17	8.8 (5.18-13.70)	<10	7.9 (1.72-21.19)	<10	10.1 (3.78-20.75)	<10	8.3 (3.61-15.83)
**Neurologic disorders**		Not shown	14.6 (9.90-20.38)	<10	12.7 (4.22-27.27)	13	22.3 (12.53-35.07)	10	10.6 (5.17-18.61)
**Renal disorders**		Not shown	1.5 (0.31-4.46)	<10	NA	<10	3.4 (0.42-11.75)	<10	1.0 (0.02-5.71)
**Immunosuppressive conditions**		11	5.8 (2.92-10.14)	<10	2.8 (0.09-13.77)	<10	8.4 (2.76-18.57)	<10	5.4 (1.80-12.20)
**Blood disorders, including sickle cell disease**		8	4.1 (1.75-7.91)	<10	2.5 (0.06-13.44)	<10	3.3 (0.38-11.56)	<10	5.2 (1.66-11.76)
**Sickle cell disease**		Not shown	1.0 (0.12-3.68)	<10	NA	<10	NA	<10	2.1 (0.24-7.38)
**Rheumatologic/ Autoimmune disease**		Not shown	0.5 (0.01-2.80)	<10	NA	<10	1.6 (0.03-8.92)	<10	NA
**Clinical outcomes and interventions**	**Length of Stay in days (Median IQR)**	192	1.9 (0.81-3.83)	39	1.7 (1.00-3.08)	59	2.8 (1.18-4.66)	94	1.6 (0.62-3.38)

[j] Calculation for obesity is determined by documented medical history or documented measurement of body mass index in the medical chart and excludes children <2 years old.

© 2025 Anglin O et al. *JAMA Network Open.*

		Total		American Indian or Alaska Native (AI/AN)		Multiracial		Unknown	
	**ICU**	Not shown	27.3 (21.10-34.15)	<10	18.2 (7.71-33.83)	24	40.3 (27.75-53.90)	22	23.0 (14.92-32.79)
	**Mechanical ventilation**	14	7.3 (4.03-11.90)	<10	5.4 (0.73-17.77)	<10	6.8 (1.87-16.43)	<10	8.3 (3.62-15.85)
	**Death**	Not shown	0.5 (0.01-2.85)	<10	NA	<10	NA	<10	1.0 (0.02-5.74)
**COVID-19 vaccination status[k]**	**No record of COVID-19 vaccination**	89	72.7 (63.88-80.43)	18	69.6 (48.55-85.91)	32	72.1 (56.59-84.58)	39	74.8 (60.82-85.80)
	**Received at least 1 dose, but not up-to-date**	24	19.7 (13.03-27.87)	<10	26.6 (11.37-47.47)	<10	18.8 (8.61-33.40)	<10	17.0 (8.00-29.96)
	**Up-to-date**	9	7.6 (3.52-13.93)	<10	3.8 (0.09-19.59)	<10	9.1 (2.52-21.65)	<10	8.2 (2.36-19.46)

[k] Children aged <6months are not eligible to receive COVID-19 vaccine and are excluded from these calculations. COVID-19 vaccination eligibility changed by age group over time during the study period. Vaccine-eligible children and adolescents ages ≥6 months without any evidence of ever having received a COVID-19 vaccination were categorized as having no record of COVID-19 vaccination. Before the 2022–2023 (bivalent) formula doses were recommended, children and adolescents who completed a COVID-19 primary series ≥14 days before the positive SARS-CoV-2 test associated with their hospitalization were categorized as "up-to-date." After the 2022–2023 booster doses were recommended, only those who completed the COVID-19 primary series and subsequently received a recommended booster dose ≥14 days before testing positive for SARS-CoV-2 were categorized as "up-to-date". Children and adolescents who began, but did not complete, a COVID-19 primary series, or who completed a primary series but did not receive a recommended booster dose ≥14 days before testing positive for SARS-CoV-2 were categorized as having "received at least one dose of COVID-19 vaccine but were not "up-to-date".

© 2025 Anglin O et al. *JAMA Network Open.*
