# Supplementary material for: Pediatric COVID-19 Hospitalization Trends by Race and Ethnicity, 2020–2023
Source: JAMA Netw Open. Author manuscript; Available in PMC 2026 Jan 27. (PMC12842959; doi:10.1001/jamanetworkopen.2025.21009)
Supplement: Supplement 2 (Data Sharing Statement) — Data Sharing Statement [file NIHMS2134914-supplement-Supplement_2__Data_Sharing_Statement_.pdf]

Data Sharing Statement

Anglin. Pediatric COVID-19 Hospitalization Trends by Race and Ethnicity, 2020-2023. *JAMA Netw Open*. Published July 15, 2025. doi:10.1001/jamanetworkopen.2025.21009

Data
Data available: No

Additional Information
Explanation for why data not available: To protect confidentiality, raw data cannot be publicly shared. Contact the corresponding author with any data-related questions.
